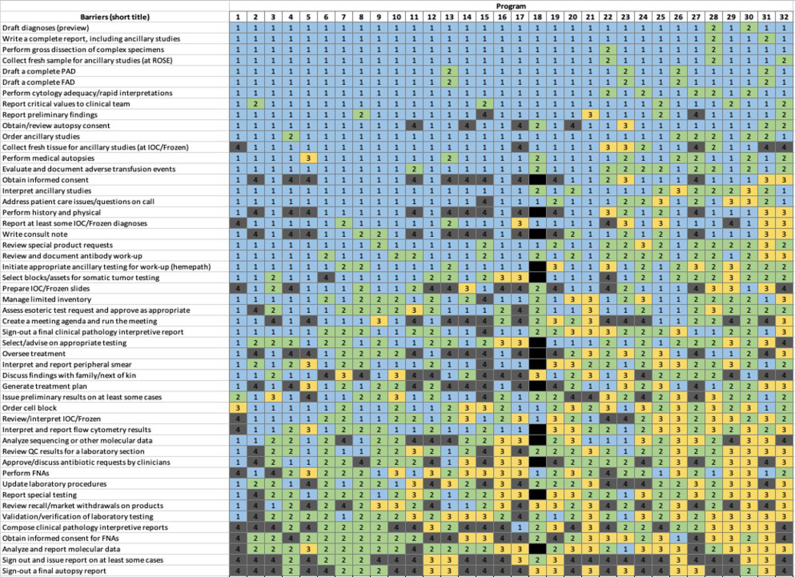# Barriers to achieving graduated responsibility: preparing pathology residents for independent practice

**DOI:** 10.1016/j.acpath.2025.100221

**Published:** 2025-09-22

**Authors:** Bronwyn H. Bryant, Balaram Puligandla, Hailey L. Gosnell, Kristen Johnson, Stephanie Barak, Mary P. Berg, Satyapal Chahar, John M. Childs, Julie Katz Karp, Amanda Lofgreen, Cindy B. McCloskey, Barbara E.C. Knollmann-Ritschel, Kristie L. White, W. Stephen Black-Schaffer

**Affiliations:** a Department of Pathology and Laboratory Medicine, University of Vermont, Burlington, VT, USA; b Department of Pathology, Oregon Health & Science University, Portland, OR, USA; c Cleveland Clinic Foundation, Department of Pathology, Cleveland, OH, USA; d CAP Learning, College of American Pathologists, Northfield, IL, USA; e Department of Pathology and Laboratory Medicine, Women and Infants Hospital, Warren Alpert Medical School of Brown University, Providence, RI, USA; f University of Colorado, Anschutz Medical Campus, Department of Pathology, Aurora, CO, USA; g University of Mississippi Medical Center, Department of Pathology, Jackson, MS, USA; h Geisinger Medical Center, Danville, PA, USA; i Department of Pathology and Genomic Medicine, Thomas Jefferson University Hospital, Philadelphia, PA, USA; j The Department of Pathology, University of Oklahoma Health Sciences Center, Oklahoma City, OK, USA; k Department of Pathology, Uniformed Services University of the Health Sciences, Bethesda, MD, USA; l Department of Laboratory Medicine, UCSF Medical Center, San Francisco, CA, USA; m Department of Pathology, Massachusetts General Hospital and Harvard Medical School, Boston, MA, USA

**Keywords:** Graduated responsibility, Medical education, Oversight supervision, Pathology residency

## Abstract

Allowing pathology residents to practice with high levels of autonomy helps prepare them for independent practice. The Accreditation Council for Graduate Medical Education (ACGME) recently added to the core requirements that residents must have opportunities to perform assigned clinical responsibilities under oversight supervision—defined as review and feedback after care is delivered. To understand the current state of resident autonomy, pathology residency programs were surveyed (32 responses) about the highest level of supervision achieved by senior residents for common pathology tasks, as well as the most significant barrier to achieving oversight supervision for each task. Each task was assigned a “supervision score” based on a weighted average of the supervision level achieved across programs. Barriers were grouped into common themes, such as “presumed patient impact,” “billing/privileges,” and “curricular structure.” Although there was consensus around achievable supervision levels for some tasks (e.g., almost all programs allowed residents to gross with oversight supervision; none allowed residents to sign-out the final reports with oversight supervision), for most tasks, the level of supervision achieved and barriers to achieving oversight supervision varied widely across institutions. This study provides insights into the challenges programs face in providing opportunities for graduated responsibility to residents. The results also suggest programs can learn from each other to achieve oversight supervision in certain tasks. Opportunities to provide more graduated responsibility are explored to achieve the ACGME requirement of “oversight supervision” to better prepare residents for independent practice.

## Introduction

A recent surge in job opportunities has cast light on the necessity of training pathologists in residency to be ready to work independently.^1^ This accentuates the long-standing issue that most pathologists practice in areas beyond subspecialty training. Residency training needs to provide preparation for practice across anatomic and/or clinical pathology, along with a variety of skills less formally taught in many training programs, such as management and administrative skills.^1^, ^2^, ^3^ Several factors result in significant deficiencies in these essential skills for new graduates, including medical school curricula providing limited exposure to pathology and residency programs not altering their rotations to allow trainees to have accountability in clinical decision-making during pathology residency training.^4^, ^5^, ^6^, ^7^, ^8^ The perceived universal need for fellowship training is both a symptom of these foundational training deficiencies and a contributor.^5^^,^^9^^,^^10^ Broader implementation of opportunities for graduated responsibility in all aspects of residency training is an important objective for training programs to allow residents to prepare for independent practice.^3^^,^^6^^,^^11^^,^^12^ The Accreditation Council for Graduate Medical Education (ACGME) core requirements now stipulate that resident experiences must include opportunities to perform assigned clinical responsibilities under oversight supervision—defined as review and feedback after care is delivered.^13^

While recent studies suggest that newly graduated pathologists are generally sufficiently prepared for practice, they have also identified several disconnects between areas emphasized in training and workforce demands.^3^ Fields often emphasized in training, but not reported to be of corresponding utility in practice, include transfusion medicine and autopsy.^3^^,^^14^ Conversely, areas often underemphasized in training and reported to be of greater value in practice include informatics, laboratory management, billing and coding, and molecular pathology. These areas of pathology tend not to be promoted as core rotations and have few direct resident learning opportunities in the typical curriculum. An examination of pathology graduate medical education literature reveals that Anatomic Pathology (AP) topics have been featured at almost twice the rate of Clinical Pathology (CP) topics,^15^ even though similar or greater training deficiencies exist in areas of CP. Optimized implementation of graduated responsibility remains essential to enhancing learning across all fields of pathology practice.

In this paper, we assess the distribution of graduated responsibility across various aspects of training in residency programs. Comparisons are made by task between programs where oversight supervision is achieved and where it is not achieved. Perceived barriers to implementation of graduated responsibility in programs not achieving resident oversight supervision are reported and analyzed.

## Materials and methods

### Survey distribution

An anonymous survey was sent to the 142 current program directors (PD) of Anatomic and Clinical pathology residencies in the US using Research Electronic Data Capture (REDCap).^16^ The survey was open for 4 weeks, and three reminder emails were sent. This study was deemed exempt from review by the University of Vermont internal review board as education-/survey-based research.

### Survey design

The survey tool asked two questions about each of 51 common AP or CP tasks: (1) What is the highest level of supervision achieved by senior residents in the program? and (2) What is the most significant barrier to allowing senior residents to practice with oversight supervision? The ACGME definitions of supervision levels were explained at the start of the survey (Table 1).^13^ If oversight supervision was achieved, instead of selecting a primary barrier, respondents could select “N/A, residents routinely achieve oversight supervision for this task” for the barrier’s question. Program Directors were encouraged to solicit input from rotation directors. A screening question ensured the respondents were PDs. Program demographic questions were included at the end.

**Table 1:** Definitions of supervision provided in survey tool.

ACGME Supervision Level	Definition and Explanation
Oversight supervision	ACGME Program Requirement VI.A.2.b). (3): The supervising physician is available to provide review of procedures/encounters with feedback provided after care is delivered. *By definition, oversight supervision implies review with the trainee after the task has been performed or the care has been delivered; supervision does not need to be immediately available during task performance or care delivery. Oversight supervision implies task is completed prior to attending review.*
Indirect supervision	ACGME Program Requirement VI.A.2.b). (2): The supervising physician is not providing physical or concurrent visual or audio supervision but is immediately available to the resident for guidance and is available to provide appropriate direct supervision *By definition, indirect supervision implies immediate availability of supervision whenever the task is performed or the care delivered, although actual review may occur afterward.*
Direct supervision	ACGME Program Requirement VI.A.2.b). (1): The supervising physician is physically present with the resident during the key portions of the patient interaction [or patient care activity]. *By definition, direct supervision occurs during performance of the task and whenever care is being delivered.*

ACGME: Accreditation Council for Grduate Medical Education.

### Supervision score

A supervision score (SS) was calculated as a weighted percentage of tasks where residents were reported to achieve oversight supervision, with half credit given if residents achieved indirect supervision. Tasks for which “N/A, activity not available” was chosen were excluded from the calculation:

$$Supervision\;Score=\frac{\left(Oversight+\frac{Indirect}{2}\right)}{Oversight+Indirect+Direct}$$

A task-specific supervision score (SS-task) was calculated for each task based on ratings across all programs (Table 2). A program-specific supervision score (SS-program) was likewise calculated for each program based on the supervision ratings of all tasks available at that program. Higher scores (closer to 1) indicate oversight supervision is more readily achieved; lower scores (closer to 0) indicate less oversight or indirect supervision is achieved.

**Table 2:** Supervision score by activity.

Anatomic pathology (AP) activity	Supervision score
**General AP**	
Draft diagnoses (preview)	0.97
Write a complete report, including ancillary studies (“simulated sign out”)	0.97
Order ancillary studies (e.g., IHC, flow cytometry, and FISH)	0.91
Interpret ancillary studies (e.g., IHC, flow cytometry, and FISH)	0.83
Issue preliminary results on at least some cases	0.58
Sign out and issue report on at least some cases	0.21
**Surgical Pathology**	
Perform gross dissection of complex specimens (CPT 88309s)	0.97
Collect fresh tissue for ancillary studies (e.g., flow cytometry, microbial culture, and cytogenetics)	0.89
Report at least some frozen section diagnoses (interpret slides and call results to surgeon)	0.79
Prepare intraoperative consultations/frozen sections (e.g., make slides)	0.70
Review/interpret intraoperative consultations/frozen sections	0.53
**Cytology**	
Collect fresh sample for ancillary studies (e.g., flow cytometry, microbial culture, and cytogenetics)	0.95
Perform cytology adequacy assessments/rapid interpretations	0.92
Order cell block	0.56
Perform FNAs	0.43
Obtain informed consent for FNAs	0.33
**Autopsy**	
Draft a complete PAD	0.94
Draft a complete FAD	0.94
Obtain/review autopsy consent	0.91
Perform medical autopsies (prosection and sampling)	0.86
Discuss findings with family/next of kin	0.60
Sign-out a final autopsy report	0.18

**Clinical Pathology (CP) Activity**	**Supervision Score**

**General CP**	
Report preliminary findings (e.g., blasts on smears, blood parasites, joint crystals)	0.92
Report critical values to clinical team	0.92
Address patient care issues/questions on call	0.82
Sign-out a final clinical pathology interpretive report (e.g., blood smear review, electrophoresis, and ANA)	0.65
Compose clinical pathology interpretive reports (e.g., blood smear review, electrophoresis, and ANA, coagulation)	0.34
**Laboratory Management**	
Assess esoteric test request and approve as appropriate	0.67
Create a meeting agenda and run the meeting	0.65
Review QC results for a laboratory section (including proficiency testing results)	0.47
Update laboratory procedures	0.42
Validation/verification of laboratory testing	0.37
**Blood** B**ank**	
Evaluate and document adverse transfusion events	0.84
Review special product requests	0.77
Review and document antibody work-up	0.73
Manage limited inventory (e.g., platelet approval)	0.68
Review recall/market withdrawals on products	0.39
**Apheresis**	
Obtain informed consent	0.83
Perform history and physical	0.81
Write consult note	0.79
Oversee treatment	0.60
Generate treatment plan	0.59
**Microbiology**	
Analyze sequencing or other molecular data	0.48
Approve/discuss antibiotic requests by clinicians	0.43
Report special testing	0.40
**Genomics/**M**olecular**	
Select blocks/assets for somatic tumor testing	0.71
Select/advise on appropriate testing	0.62
Analyze and report molecular data	0.30
**Hematopathology**	
Initiate appropriate ancillary testing for bone marrow or lymphoma work-up	0.73
Interpret and report peripheral smear	0.60
Interpret and report flow cytometry results	0.53

ANA: anti-nuclear antibody; FAD: final autopsy diagnosis; FISH: fluorescent in situ hybridization; FNA: fine-needle aspiration; IHC: immunohistochemistry; PAD: preliminary autopsy diagnosis; QC: quality control; ROSE: rapid on-site evaluation.

### Statistical analysis

Because the data were anonymous, the institutions they represent were not identifiable. Descriptive statistics were computed using IBM SPSS Statistics for Windows, version 25.0 (IBM Corp, Armonk, New York). All other statistical analyses were performed using Microsoft Excel for Microsoft 365.

## Results

### Response rates

Thirty-nine of 142 programs (27%) started the survey, and 32 programs (22.5%) provided responses to at least 70% of the activities; these latter were included in the analysis. Only 30 programs (21%) responded to demographic questions (Table 3).

**Table 3:** Program demographics.

Program characteristic	N (%)
**Programs with responses (n/total programs)**	39/142 (27%)
**Programs with responses to >70% of activities (n/total responses)**	32/39 (82%)
**Program size (N = 30)**	
9–15 residents	6 (20%)
16–23 residents	15 (50%)
24–31 residents	5 (17%)
32–39 residents	3 (10%)
40 or more residents	1 (3%)
**Program location (N = 30)**	
New England	5 (17%)
Mid-Atlantic	4 (13%)
East North Central	2 (7%)
West North Central	4 (13%)
South Atlantic	3 (10%)
East South Central	1 (3%)
West South Central	5 (17%)
Mountain	1 (3%)
Pacific	5 (17%)
**Competition between resident and fellow? (N = 30)**	
Yes	1 (3%)
No	24 (80%)
Sometimes^a^	5 (17%)

a Rapid on-site evaluation/fine-needle aspiration most commonly cited activity.

### Intercorrelations among barriers to oversight supervision

Table 4 presents the intercorrelations among oversight supervision and teaching barriers. Statistical significance was examined using a Bonferroni-corrected α level of 0.002 per test (0.05/21). Results reveal two trends. A significant positive correlation was noted between two pairs of barriers, specifically between “irreversible impact to patient care” and “faculty resistance to allowing practice with oversight supervision” and between “hospital privileges” and “billing.” The first pair made logical sense to the authors as “faculty resistance to allowing practice with oversight supervision” may plausibly stem from a fear of “irreversible impact to patient care.” Thus, given the high degree of correlation of these responses, they were combined in subsequent analyses under the designation of “presumed patient impact.” Likewise, hospital privileges and billing constraints represent related regulatory or administrative factors beyond the control of most programs, and so these were similarly combined in subsequent analyses under the designation of “privileges/billing.”

**Table 4:** Intercorrelations of oversight supervision and teaching barriers.

	Routinely attains oversight	Irreversible impact	Faculty resistance	Hospital privileges	Billing	Curriculum not structured
**Irreversible impact to patient care**	−0.43					
**Faculty resistance to allowing practice with oversight supervision**	−0.25	0.50∗				
**Hospital privileges**	−0.64∗	−0.11	−0.20			
**Billing**	−0.41	0.01	−0.41	0.67∗		
**Curriculum is not structured for oversight supervision in this area**	−0.65∗	−0.16	−0.13	0.03	−0.07	
**Not all residents are entrustable for oversight supervision**	−0.40	0.01	0.16	−0.15	−0.12	0.36

∗P < 0.002.

After combining these two pairs of highly correlated barriers, we see a significant negative correlation between oversight supervision and three barriers: “presumed patient impact,” “privileges/billing,” and “curricular structure” (Table 5). Statistical significance was examined using a Bonferroni-corrected α level of 0.005 per test (0.05/10).

**Table 5:** Intercorrelations of oversight supervision and grouped barriers.

	Routinely attains oversight	Presumed Patient Impact	Privileges/Billing	Curricular Structure
Presumed Patient Impact	−0.42∗			
Privileges/billing	−0.48∗	0.09		
Curricular Structure	−0.65∗	−0.01	−0.04	
Not Entrustable	−0.40	0.02	−0.11	0.36

∗P < 0.005.

### Supervision score by task

**Fig. 1:**
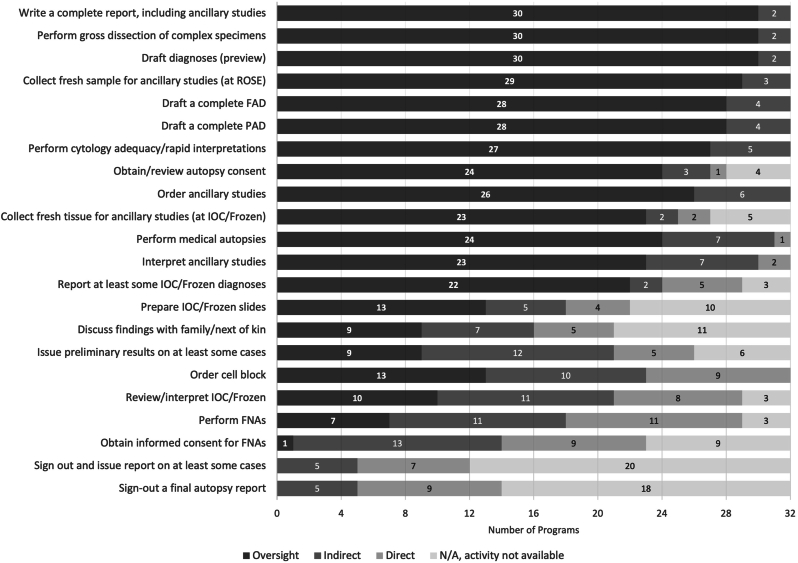
Highest level of supervision achieved for AP tasks. Tasks are sorted by task supervision score (highest to lowest). AP: anatomic pathology; FAD: final autopsy diagnosis; FNA: fine-needle aspiration; IOC: intraoperative consultation; PAD; preliminary autopsy diagnosis; ROSE: rapid on-site evaluation.

**Fig. 2:**
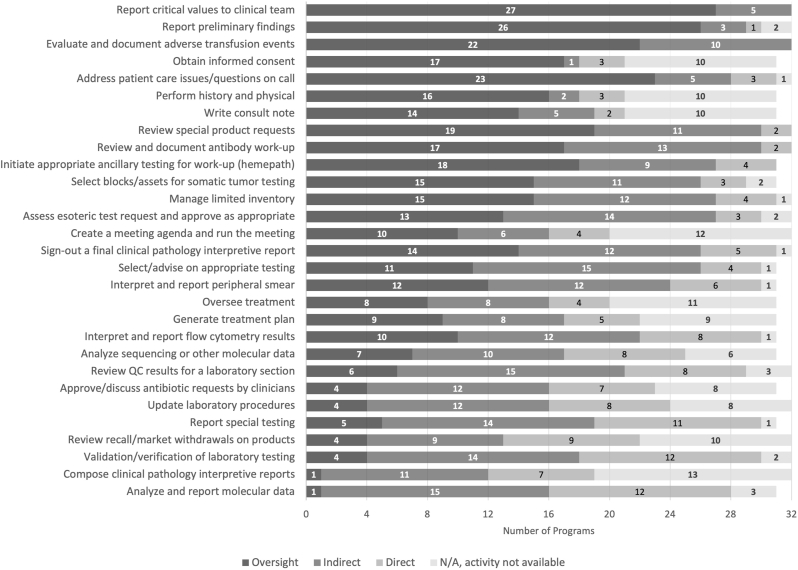
Highest level of supervision achieved for CP tasks. Tasks are sorted by task supervision score (highest to lowest). CP: clinical pathology; QC: quality control

Entrustment is the act of placing trust in someone to appropriately handle a specific task or situation. The highest level of entrustment achieved for AP and CP, ordered by descending SS-task, shows some expected trends (Fig. 1, Fig. 2). Most AP tasks (14/20) achieved oversight supervision at >50% of the programs, with almost all programs reporting residents achieving oversight supervision for grossing and drafting diagnoses. There are two tasks where none achieved oversight supervision: “sign-out and issue report on at least some cases” and “sign-out a final autopsy report.” Many programs cited these activities as not available to trainees at their program.

For CP tasks, notable trends include higher percentage of programs achieving oversight supervision for tasks that are required by regulatory agencies and/or may happen after business hours (e.g., “report critical values to clinical team” and “evaluate and document adverse transfusion events”). A few physician-specific tasks are also included (e.g., “perform history and physical”). Tasks that generate a pathology report have a lower SS-task (“composed a clinical pathology interpretative report” and “analyze and report molecular data”); however, the division of duties between residency and fellowship training on the latter topic is uncertain. Lower SS-task scores were also noted for laboratory management-oriented tasks (e.g., “validation/verification of laboratory testing” or “update laboratory procedures”).

No CP task had a preponderance of “N/A, activity not available” responses, but tasks with this designation more frequently included lab management topics such as “validation/verification of laboratory testing” (n = 13) and “create a meeting agenda and run a meeting” (n = 12), followed by the four apheresis topics.

Except for the final reporting of cases in AP, each task had at least one program reporting that residents were routinely achieving oversight supervision.

### Barriers to oversight

**Fig. 3:**
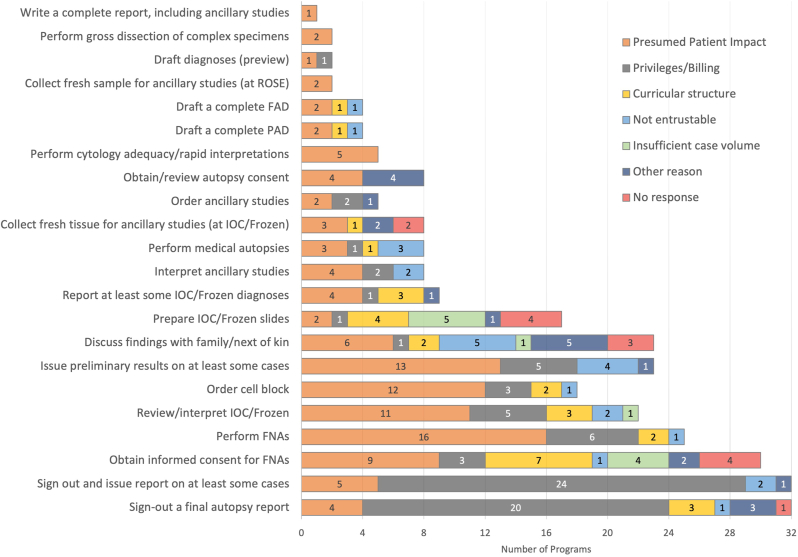
Barriers to achieving oversight supervision for AP tasks. Tasks are sorted by task supervision score (highest to lowest). Includes responses of “indirect supervision,” “direct supervision,” and “N/A, activity not available.” AP: anatomic pathology; FAD: final autopsy diagnosis; FNA: fine-needle aspiration; IOC: intraoperative consultation; PAD; preliminary autopsy diagnosis; ROSE: rapid on-site evaluation.

**Fig. 4:**
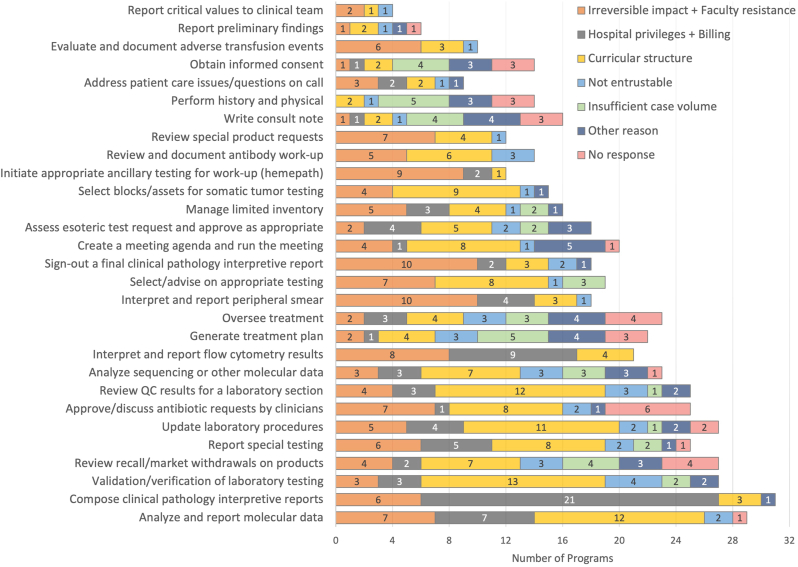
Barriers to achieving oversight supervision for CP tasks. Tasks are sorted by task supervision score (highest to lowest). Includes responses of “indirect supervision,” “direct supervision,” and “N/A, activity not available.” CP: clinical pathology; QC: quality control.

Barriers to achieving oversight supervision for AP and CP tasks, ordered by descending SS-task, also exhibit some expected trends (Fig. 3, Fig. 4). For all AP tasks, “presumed patient impact” was reported as a barrier by at least one program. The tasks where this was most commonly cited were the cytology tasks of “Perform fine-needle aspiration (FNA)” (n = 16), “Order cell block” (n = 12), and “Obtain informed consent for FNAs” (n = 9), “Perform cytology adequacy/rapid interpretations” (n = 5); autopsy tasks of “Discuss findings with family/next of kin” (n = 6), “Sign-out a final autopsy report” (n = 4), “Obtain/review autopsy consent” (n = 4); frozen section tasks “Review/interpret intraopertive consultation/Frozen” (n = 11), “Report at least some IOC/Frozen diagnoses” (n = 4), and general AP tasks of “Issue preliminary results on at least some cases” (n = 13), “Sign out and issue report on at least some cases” (n = 5), and “Interpret ancillary studies” (n = 4).

For the two AP tasks where no program reported achieving oversight supervision, over half of programs cited “privileges/billing” as the barrier (“sign out and issue report on at least some cases” [n = 24] and “sign-out a final autopsy report” [n = 20]). Many of these responses did not even consider these tasks achievable with indirect supervision but simply stated “N/A, activity not available at this program,” suggesting a conceptual barrier to residents performing this task in residency.

Infrequently, barriers for AP tasks included “curriculum not structured for oversight supervision” or “insufficient case volume” for tasks such as “obtain informed consent for FNAs” (n = 7) and “prepare IOC/frozen slide” (n = 4). Residents were deemed “not entrustable” to “discuss autopsy findings with next of kin” (n = 5) and to “issue preliminary results on at least some findings” (n = 4). Rarely, programs cited “other reasons” or provided no response for a barrier to a task.

For CP tasks, “presumed patient impact” and “curricular structure” were more commonly reported for many tasks. Tasks with a reporting element often cited “presumed patient impact” as a barrier, along with tasks more directly tied to patient treatment or laboratory expenditure (e.g., “approve/discuss antibody requests by clinicians” and “review special products request”). Many of the laboratory management–oriented tasks cited “curricular structure” as a barrier to achieving oversight. This is a notoriously difficult skillset to teach in residency as day-to-day responsibilities may be the primary responsibility of laboratory supervisions. “Privileges/billing” was the most common barrier for tasks that generated a pathology report (“compose a clinical pathology interpretative report”), mirroring analogous AP tasks.

Primarily for tasks related to apheresis, which is not available at every training program, 4–5 programs cited “insufficient case volume” as a barrier. Overall, “not entrustable” and “other reasons” were infrequently cited barriers for most CP tasks.

Of note, responses were slightly confounded by two programs who selected “indirect supervision” as the highest achievable level of autonomy for multiple tasks but for a barrier selected “N/A, residents routine achieve oversight in this activity”. While these options were intended to be mutually exclusive, the survey was not designed to exclude such responses. Therefore, these program responses are counted as “indirect supervision” (Fig. 1, Fig. 2) but not included in the figure of “barriers” (Fig. 3, Fig. 4), resulting in a slight numerical discordance.

### Supervision score by program

**Fig. 5:**
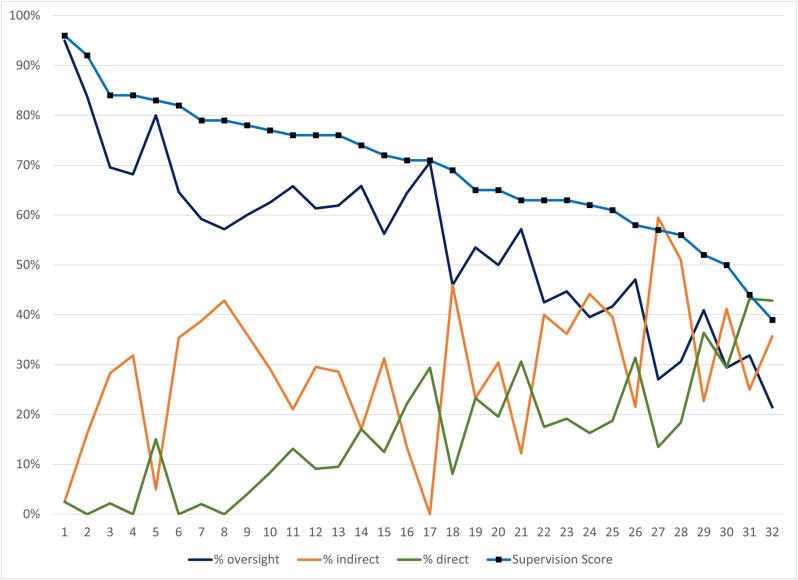
Program Supervision Scores plotted with percentage of activities achieving oversight, indirect, and direct supervision.

Individual programs were given an overall SS, which is plotted with the percentage of tasks achieving oversight, indirect, and direct supervision (Fig. 5). As programs are sorted by SS-program, this shows that responding programs represent a spectrum from those that allow more resident autonomy to those that are more restrictive in resident activities. In general, indirect supervision is higher than direct supervision for most tasks. A heatmap of the supervision rating for each task by each program is provided in Supplemental Figure 1.

## Discussion

The list of tasks studied in this manuscript reflects the common tasks residents are expected to learn throughout an AP/CP residency and perform independently when they enter practice. This work builds on studies demonstrating that residents who are able to practice as close to an attending level as possible in residency are more prepared for independent practice.^14^ This is achievable as “oversight supervision” within the ACGME requirements.^13^ Identifying the perceived barriers to pathology residents achieving “oversight supervision” is important to enable the specialty to progress toward higher levels of graduated responsibility during residency.

For almost every task, at least one program said oversight supervision was achievable, which presents an encouraging prospect. Programs not yet achieving oversight supervision in any given task may be able to learn something from the curriculum or systems at other programs. We offered responding institutions the opportunity to self-identify, which provides an opportunity for follow-up investigation to identify possible best practices for eliminating or mitigating barriers. Interviews with selected programs to explore these opportunities are in progress.

Two AP tasks notably had no programs achieving oversight supervision: “sign-out and issue report on at least some cases” and “sign-out a final autopsy report.” Over half of programs reported these tasks being unavailable to residents at their institution with “privileges/billing” as the most common barrier. This reflects the current practice pattern in pathology, whereby a resident’s preliminary results are deemed inappropriate to be reported to the chart. While not surprising, this pattern is of concern when viewed through the lens of preparing residents for independent practice. The anxiety of “clicking the button” for the first time is well described in new-in-practice pathologists.^17^ Mass General Brigham’s “Promotion-in-Place” pilot program has achieved independent practice including reporting, and their experience is a big step forward for pathology practice.^18^ Radiology has been able to incorporate issuing preliminary results into their core requirements,^19^ with several studies reporting very low discrepancy rates on overnight preliminary interpretations.^20^, ^21^, ^22^ Pathology may learn something from a field that similarly delivers most patient care via written reports. For programs who reported achieving “indirect supervision” for reporting tasks, understanding how to move more programs to this level is necessary.

Certain laboratory sections demonstrated themes to supervision levels achieved and barriers reported. Clinical pathology has a higher proportion of management-/administrative-related tasks and fewer case-based activities. Laboratory management topics, a challenging topic to teach residents,^15^ appeared more often as “activity not available” for reasons such as “curricular structure.” Teaching laboratory management in day-to-day activities is challenging, and programs that offer longitudinal experience over the course of several months to a year may have more success in providing such experiences to residents.^23^, ^24^, ^25^ Perhaps it is time for pathology to embrace the idea of a longitudinal model like a “continuity clinic” to provide exposure to such non–case-based experiences. Examples include assistant medical directorship experiences, but little is published on such curricular elements. Other activities, such as apheresis tasks, were not available due to “insufficient case volume” or “curricular structure,” where the apheresis service may be unavailable or run by a different department at that hospital.

Tasks where “resident is not entrustable for this activity” may imply oversight supervision would be achievable, but from the data, it was not clear if all or only some residents were considered not entrustable. This may merely illustrate a conceptual inconsistency in how residency is viewed—residents are in-training, and, therefore, not entrustable for tasks considered to be attending-level responsibilities; however, if the program were to graduate residents who were not entrustable for core clinical responsibilities, this would be an educational failing. Some programs may offer higher autonomy at the fellowship level. Even if a resident has the knowledge and skills to perform certain tasks, they might not be allowed to perform them because those tasks are preserved for the fellows. This structure precludes opportunities to achieve and assess general competency of higher-performing residents prior to graduation. Such possibilities need to be explored in follow-up interviews with programs.

The 2024 American Society for Clinical Pathology (ASCP) annual fellowship and job market survey (conducted as part of their resident in service examination) reported only 2% of residents in training not intending to complete a fellowship before applying for a job.^26^ In contrast, the 2024 and 2023 ABPath practicing and training surveys of new in-practice pathologists (conducted in conjunction with their Continuing Certification program) reported 13–18% of the respondents having not completed a fellowship (personal communication, Dr. Gary Procop, CEO, ABPath). The ABPath Continuing Certification survey includes pathologists who completed training in the past 10 years. This difference between residents’ intent to complete a fellowship contrasted with fellowships actually completed is important to keep a close eye on as it strongly supports residents needing to achieve entrustment in core tasks by the end of residency in preparation for practice—another reason our profession cannot postpone “oversight supervision” to fellowship training.

Comparing SSs across programs presented an intriguing distribution. While this spectrum of programmatic supervisory scores necessarily relates to the culture of each program, achieving consistency across programs in the overall entrustment level of the residents has implications for the specialty as a whole. The qualities of a “permissive” program—how they balance autonomy with safe patient care—are an essential area for investigation. Were the profession able to achieve consensus on tasks for which it is appropriate for a resident to achieve oversight supervision in residency, individual programs would have a benchmark against which to assess their residency program and an evidence base for targeting improvements in their curriculum to achieve graduated responsibility. Such a project would be valuable to pursue.

This study also provides a framework for individual residents to discern opportunities in which they can demonstrate their capabilities and advocate for programmatic changes to maximize their autonomy in training. Some examples of situations in which residents can demonstrate additional capacity for autonomy include asking to order stains on cases prior to review with the attending, pursuing a case and reporting to completion before handing off to the attending for sign-out, and providing an interpretation of a rapid on-site evaluation (ROSE)/frozen section before the attending speaks. If residents can take the lead in maximizing available learning opportunities within the confines of their program, thereby demonstrating the extent of their abilities, they can play an active role in advocating for training system reform. Alternatively, if a program does not have the case material or resources to offer more graduated responsibility (especially in an area of high interest to the resident), the resident can advocate to do an elective to gain such experiences.

On December 6, 2024, Center for Medicare and Medicaid Services (CMS) released revised regulations with interpretive guidelines for laboratories. These interpretive guidelines qualified residency graduates who are in the process of obtaining board certification but excluded graduates who are neither certified nor in the process of seeking certification. In the authors’ opinion, this action on the part of CMS emphasizes the importance of ensuring that residency programs enable residents to experience and develop appropriate confidence in the essential aspects of practice while still in training and likewise enable PDs to assess their residents’ readiness for progression to independent practice as early and accurately as possible. Other specialties have summarized entrustment-based assessment data to understand resident readiness for practice, and the results suggest programs are graduating a subset of residents they do not feel are ready for independent practice.^27^ To our knowledge, such a question has not been explicitly posed to PDs of pathology residency or fellowship programs, although there is information on preparation for practice of new-in-practice pathologists in the academic and private sectors^17^ and new-in practice physicians in other fields.^28^^,^^29^ Such a study would be informative to monitor the impact of any increase in oversight supervision in pathology residency.

Limitations of this study include recall bias by PDs or their limited knowledge of graduated responsibility in practice areas outside their individual scope of practice. This was mitigated by providing a printable version of the survey to gather input from rotation directors. Programs were asked to identify the most significant barrier to achieving oversight supervision, and for many tasks, there may be more than one barrier in play. Furthermore, while PDs were asked to reflect on the most senior residents as a whole, decisions about graduated responsibility should be made on an individual basis. Lastly, there are no established national benchmarks for what a trainee should be able to do at the end of training, and training programs vary widely in their circumstances, including local institutional policies and state laws.

We present the level of supervision achieved across 32 residency programs for the common tasks of residency and explore the barriers to achieving oversight supervision. As there is considerable variability from program to program, there are opportunities to learn from each other about how to provide graduated responsibility across residency programs. To achieve major changes and national uniformity, efforts are needed at the level of AGCME requirements, which may in turn entail rethinking pathology hospital privileges at some institutions and changes in fellowship training. Job expectations require us to commence reassessment of residency training now. At the end of training, PDs must attest that residents are competent to practice, and if residents are not achieving “oversight supervision,” how can they know a resident is prepared for independent practice?

## Author’s note

For Dr. Ritschel, the opinions expressed in this educational case are those of the authors and do not reflect the official views or position of the Uniformed Services University, Department of the Navy, Department of the Army, Department of the Air Force, or the Department of Defense.

## Funding

Funding and support is provided by the College of American Pathologists in the form of educational staff support (authors KJ and AL), travel reimbursement for committee members (authors B.H.B., B.P., H.L.G., S.B., M.P.B., S.C., J.M.C., J.K.K., C.B.M., B.E.C.K.R., and K.L.W.), and funding for publication.

## Declaration of competing interest

The authors declare that they have no known competing financial interests or personal relationships that could have appeared to influence the work reported in this paper.

## Figures and Tables

**figs1:**