# Supplementary material for: Barriers to achieving graduated responsibility: preparing pathology residents for independent practice
Source: Acad Pathol. 2025 Sep 22;12(4):100221. doi: 10.1016/j.acpath.2025.100221 (PMC12493152; doi:10.1016/j.acpath.2025.100221)
Supplement: Multimedia component 1 [file mmc1.docx]

Supplemental Figure 1. Heatmap of supervision ratings for each task by each program. Ratings are coded as follows: 1/blue=oversight supervision, 2/green = indirect supervision, 3/yellow = direct supervision, 4/gray = activity not available, and black = no response.
